# Social and environmental determinants influencing injection drug use and HIV risk among two sister cities on the US–Mexico border: a comparative cross-sectional study, 2016–2018

**DOI:** 10.1186/s12954-023-00802-0

**Published:** 2023-07-03

**Authors:** Julia Lechuga, Rebeca Ramos, Natasha Ludwig-Barron, Gilberto Perez, Maria Elena Ramos, João B. Ferreira-Pinto, Jacquelin I. Cordero, John Sauceda, Jorge Salazar

**Affiliations:** 1 College of Health Sciences, The University of Texas at El Paso, El Paso, USA; 2 The University of California San Francisco, San Francisco, USA; 3 Texas State Health Department in Houston, Houston, USA; 4 Programa Compañeros, Ciudad Juárez, Chihuahua, Mexico; 5 The University of Texas Health Science Center at Houston (UTHealth) School of Public Health, Houston, USA

**Keywords:** US–Mexico border, HIV, Injection drug use, Harm reduction, Social determinants of health, Social networks, Border health, Community-based participatory research

## Abstract

The economic, social, cultural and political milieus that influence injection drug-related HIV risk behaviors along the US–Mexico border in the previous decade have been studied comparing cities on an East–West axis. In an effort to inform interventions targeting factors beyond the individual level, we used a cross-sectional study design comparing people who inject drugs during 2016–2018, living on a North–South axis, in two cities—Ciudad Juárez, Chihuahua, Mexico and El Paso, Texas, USA—situated at the midpoint of the 2000 US–Mexico borderland stretch. We conceptualize injection drug use and its antecedents and consequences as influenced by factors operating at various levels of influence. Results of analysis comparing samples recruited from each border city indicated significant differences in demographic, socioeconomic, micro- and macro-level factors that affect risk. Similarities emerged in individual-level risk behaviors and some dynamics of risk at the drug use site most frequented to use drugs. In addition, analyses testing associations across samples indicated that different contextual factors such as characteristics of the drug use sites influenced syringe sharing. In this article, we reflect on the potential tailored interventions needed to target the context of HIV transmission risk among people who use drugs and reside in binational environment.

## Background

It is now widely acknowledged that the socio-structural environment exerts a powerful influence on the natural history of substance use disorders and associated risk behaviors, predisposing people to blood-borne and sexually transmitted infections (STIs), including the human immunodeficiency virus (HIV) [1–6]. However, the bulk of intervention research studies continues to focus on individual-level factors perhaps because the vast majority of prior research conducted to inform interventions has relied on individual-level theories of behavior change [7].

Prominent health behavior theories, such as the Health Belief Model [8, 9] and the Theory of Reasoned Action [10], posit that individual-level factors account for health and disease. Interventions based on these theories primarily aim to reduce high-risk behaviors by modifying individual-level behaviors, beliefs and attitudes. Conversely, the Risk Environment Framework [4, 11, 12] incorporates socioenvironmental factors as explanations of disease transmission. Research with People Who Inject Drugs (PWID) around the world, informed by the risk environment framework, indicates that the social and physical features of environments constrict individual agency and determine engagement in behaviors that place PWID at risk of contracting infectious diseases [13–15]. It is posited that these environmental features exert their influence at the macro- or micro-levels. This classification has been made to assist researchers in distinguishing between distal and more proximal determinants of risk that are extraneous to the individual [4, 14]. It is in this context that we conceptualize drivers of behavior, as those arising from the risk environment, which Rhodes and Quirk [12] describe as the broad social structures (macro) versus immediate physical space, setting, and groups (micro) in which a variety of factors exogenous to the individual interact to increase HIV transmission risks [4, 5, 12]. However, this macro-/micro-categorization is artificial as factors interact in complex ways across the continuum [11]. A macro-level influence refers to structural-level factors extraneous to the individual such as policies, policing, and incarceration. On the other hand, micro-level factors refer to factors that may exert a more proximal influence such as the physical and social characteristics of the drug use environment such as features of the injecting environment that may facilitate or inhibit risk behaviors [16].

Geographical binational borders are regions where two countries meet. These regions are particularly appropriate environments to examine these micro-/macro-level influences, as there are often stark differences in policies, priorities, and available resources in neighboring countries. Research suggests that macro-level factors such as aggressive policing [17–19] and high population mobility due to emigration and immigration [19, 20] are central, risk enhancing, macro-level features of the US–Mexico border risk environment. Moreover, research by Ramos et al. [3] indicates that structural and social factors including cross-border mobility, arrest and incarceration of PWID by local law enforcement, housing instability, and intergenerational drug use played a key part of the drug-use-related HIV risk environment of the US–Mexico border and importantly that risk factors varied across the Mexican cities of Ciudad Juárez, Chihuahua and Tijuana, Baja California. This finding indicates that the influence of social and environmental factors on health varies according to the culture, economy, and geographical location of groups or populations, and these factors in turn shape health-related risk behaviors [7]. This finding underscores that the risk environment of seemingly similar environments may differ warranting different approaches to reduce harm.

The US–Mexico border is a ‘natural laboratory’ for studying differences in drug use risk environments as both countries differ in language, culture, and stage of economic development. The US–Mexico border is made up of six Mexican states, which are home to approximately 27 million people [21], and four US states which are home to approximately 75 million people [22]. According to the 2020 US Census Bureau, 7.5 million people reside in the 44 counties and 80 municipalities that comprise the US–Mexico border. The majority of residents reside in 15 pairs of “sister cities” [23]. Unfortunately, there is growing concern among healthcare providers along the US–Mexico border about the threats posed by drug use and its negative consequences (HIV, Hepatitis C, violence, etc.). Like other parts of the world, the HIV epidemic on the US–Mexico border is disproportionately affecting specific subpopulations such as men who have sex with men (MSM) and people who use drugs (PWUD). The US–Mexico border has recently seen substantial increases in the availability of heroin and fentanyl. These increases indicate a fast-growing supply of illicit opioids and, as a result, increases in infectious diseases, overdoses, and overdose deaths.

The twin cities of Ciudad Juárez, Chihuahua–Mexico and El Paso, Texas–U.S., (CJ/EP) make up the second largest international point of entry from Latin America into the USA. El Paso has an estimated population of more than 721,000 people and Ciudad Juárez has an estimated population of 1,512,354 people. The two cities form a metropolitan area of over 2,200,000 people, making it the second largest international border community in the world [21, 24, 25]. The cities are economically interdependent and characterized by high cross-border mobility. There are five international bridges connecting CJ/EP, which operate 24 h a day/seven days a week. During December of 2021, there were one million personal vehicles and half a million pedestrian crossings [26].

El Paso is situated at the far west end of the state of Texas bordering New Mexico to the West and Cd. Juarez, Chihuahua, Mexico to the south [23]. In El Paso, more than 80% of the total population are people of Latin American descent, with 70% of the population speaking Spanish [24]. Populations of Latin American descent are highly marginalized with very limited healthcare coverage [27]. El Paso is one of the poorest counties in Texas and the nation where 31% of residents live below the federal poverty line and approximately 40% rely on government sponsored healthcare insurance [24]. Additionally, the opioid crisis on the US side of the border has been growing steadily since 2005 and now includes substantial increases in the availability of heroin and fentanyl. Overdose deaths involving heroin more than doubled from 214 in 2007 to 569 deaths in 2017. Overdose deaths involving fentanyl tripled from 118 to 348 deaths during the same period.

Located in the Mexican state of Chihuahua, Ciudad (Cd.) Juárez, Mexico, is located approximately midpoint along the border between Mexico and the USA and is part of a 2-million people metroplex including the cities of El Paso in Texas and Las Cruces in New Mexico [28]. Approximately 24.4% of residents do not have access to healthcare services and 37.7% are living below the poverty level [16]. A 2001 study using a capture-recapture methodology estimated that there were approximately 6000 PWID and as many as 186 shooting galleries in Cd. Juárez [29]. Drug use in Cd. Juarez has increased dramatically alongside the explosion of violent crime experienced in the region within the past decade. Violent crime has also caused changes in the context of drug use in the region, including an increase in abandoned buildings used as drug use and sex work locations. The situation has become more critical with a recent influx of thousands of migrants from South and Central America detained across the US–Mexico border.

Our study aim is to elucidate the micro- and macro-level factors that shape the risk environment and influence the HIV risk of PWID in the sister cities of CJ/EP, which may differentially influence the spread of HIV and related blood-borne infections. The present study was conducted between 2016 and 2018, and immediately prior to and during this period two historical events occurred at the US–Mexico border, which have altered the risk environment. The first event is the unprecedented level of violence because of drug cartel wars and militarized policing that took place between 2008 and 2014 in CJ. Approximately 10,000 murders were reported in CJ between 2008 and 2012 during the peak of the violence. In 2010, the homicide rate was 471.9 per 100,000 inhabitants among males between 30 and 44 years of age compared to the national average of 59.7 [30]. Research suggests that violence radically shaped the risk environment in both CJ and EP [31]. Since 2011, the region has continued to experience peaks in violence. More recently, in 2020 the crime rate increased by 42%, with > 650 murders related to organized crime infighting in the region [32]. The second event refers to the unprecedented number of immigrants arriving in CJ starting in October 2018. Approximately 7000 immigrants traveling in a caravan, destined to the US and traveling from Central America to Mexico, arrived at the US–Mexico border. Presently, it is estimated that approximately 6000 immigrants from Cuba and Latin America, some who have been deported from the USA and some who are awaiting to cross into the USA, are living in CJ. Moreover, research indicates that recently arrived immigrants are facing dire conditions that have led them to enter the sex work trade and engage in substance misuse to cope with their living situation [33]. The characteristics of the risk environment unveiled in this study are likely to reflect the changing conditions of the risk environment due to violence and population mobility.

## Methods

### Study context: a binational community–academic partnership and study population

To strengthen community resources and address HIV and drug use in EP/CJ region, we undertook a community-based participatory research (CBPR) study targeting people who were actively using illicit substances (e.g., heroine and/or crack cocaine). Formally entitled, *Project Encuentro*, this collaboration included binational community-based organizations, researchers, and peers to adapt and implement a harm reduction intervention with the ultimate goal of assessing and reducing the HIV risk of persons who use drugs in the twin cities of CJ/EP [34]. Project Encuentro consisted of two phases: (phase I) assessment of risk factors and (phase II) implementation and testing of a social network intervention to increase HIV testing, a peer network behavioral intervention to reduce sexual and drug use risk, and community-wide events targeting structural factors affecting HIV risk. The data presented in this manuscript were derived from phase I which consists of two cross-sectional baseline surveys administered prior to intervention roll-out during years 2016–2018. We followed the STROBE (Strengthening the Reporting of Observational Studies in Epidemiology) statement reporting checklist for this cross-sectional study [35].

The eligibility criteria for survey participation included being at least 18 years, residing in either CJ/EP, having used heroin and crack/cocaine within the previous month, and being able to provide informed consent. We employed respondent-driven sampling to recruit participants. This method was chosen based on the extensive evidence that supports its use to recruit “hidden populations”. This approach incorporates chain-referral sampling and structured incentives [36]. Outreach workers recruited potential “seeds” from target communities [37]. “Seeds” were individuals who are peers and are well known and trusted in the community of people who use drugs (PWUD). Participants who qualified as seeds and volunteered to participate were provided with three coupons to recruit members of their social network into the study. The coupons were wallet size paper cards that specified the location for eligibility criteria verification and included the ID of the recruiter. Seeds visited places where people who use drugs congregate such as shooting galleries, motels, and bars and approached potential participants privately to inquire about interest in participating in a study. If potential participants conveyed interest, seeds explained the study and administered a short questionnaire to verify eligibility criteria. Seeds were asked to give the coupons to three of their peers who use drugs to be screened for eligibility by outreach staff in a location of their choice. Participants who qualified and volunteered for participation were consented and interviewed in a private location. The survey lasted between 40 and 60 min to complete. Research staff read survey questions face to face and recorded responses. After eligible participants recruited by the seeds answered the survey, they were asked to recruit from their own social network. Participants were then compensated with $10 and received $5 for each participant they successfully recruited to the study. All participants were offered an HIV test and harm reduction supplies after completing the survey. The study was approved by The University of Texas at El Paso Institutional Review Board (IRB) and the Universidad Autonoma de Chihuahua IRB board.

### Survey content

Surveys assessed demographic characteristics and socioeconomic factors (gender, marital status, income, employment, education, sources of income, and medical insurance). In addition, the survey assessed engagement in behaviors that are known risk factors for HIV infection such as condomless sex and history of sexually transmitted infection diagnoses.

Surveys assessed the type of drug use site most frequented by participants. Participants were provided with a list of possible drug use venues including at home, shooting galleries, public spaces such as public restrooms and asked to select the type of drug use venue where they had used drugs most frequently in the last 30 days. Participants were then asked to identify the dynamics of risk of the drug use site most frequented to use drugs in the last 30 days and to indicate whether drugs are available for purchase, whether individuals engage in polysubstance use, syringe sharing, and sex for drug exchanges when consuming at the site. Response options were dichotomous 0 = ‘No’ and 1 = ‘Yes’. Individuals were also asked to respond to two questions about the frequency with which they injected with others or alone while using drugs in the last 30 days. The response options were captured on a Likert-type scale ranging from 0 = ‘Never’ to 5 = ‘Always’. These variables are factors that exert an effect at the micro -level because they influence risk through the features of the immediate drug use space and through interpersonal social influence including norms and peer pressure [4].

The survey also contained variables that assessed macro-level factors associated with the sociocultural and socioeconomic context, such as unstable housing and substance use stigma. Participants were asked whether they had stable housing 0 = ‘No’ and 1 = ‘Yes’. To assess drug use stigma, participants answered six items that assessed the extent to which participants felt stigmatized due to their substance use. A sample item is “people reject me because I use drugs” and response options were captured on a Likert-type scale ranging from 1 = ‘Never’ to 4 = ‘All the time’. These factors operate at the societal level and are considered the result of broad social structural characteristics of a setting [4].

### Statistical analysis

We compared micro-, macro-, and behavioral characteristics by city of recruitment. Specifically, Chi-square tests were computed to compare categorical variables, and t-tests were used to compare continuous variables, where appropriate. Two general linear mixed model (GLMM) equations specifying a binomial distribution with a logit link function were computed to assess the influence of factors assessed on syringe sharing for each of the cities. We specified a binomial distribution as the dependent variable, syringe sharing, was dichotomous (0 = ‘No’ and 1 = ‘Yes’). To account for data non-independence because of the recruitment strategy that was employed a variable capturing the nestedness of the data created by the recruitment strategy was included as a random effect in the equations (38, 39). Variables that emerged as significantly different between sites were entered in each equation. To reduce multi-colinearity we selected only one variable of several that were strongly related to each other. For example, if profiles in education, employment, and medical insurance status were different across cities, we selected only one of these to include in the corresponding equation. The aim of computing GLMM equations was to understand which factors would emerge as significantly associated with syringe sharing when a mix of variables capturing factors at various levels of influence that distinguish between city environments is included in the equation.

## Results

A total of *N* = 363 (El Paso [*n* = 187]; Cd. Juarez [*n* = 176]) respondents met criteria and were included in the final analysis. Tables 1, 2, 3, 4 and 5 present comparisons across cities in domains of risk operating at various levels of influence. Table 1 presents comparisons of samples across demographic and socioeconomic factors.

**Table 1:** Demographic and socioeconomic characteristics

Demographics	Total *N* = 363	El Paso *N* = 187	Ciudad Juarez *N* = 176	Z (95% CI of the difference)	*P* -value
*Percent unless indicated*					
Female	20	24.6	15.4	− 2.17 (− .17,− .009)	0.03
Partnered	30.5	22.6	39.2	3.42 (.07,.258)	0.001
*Socioeconomic factors*					
Completed secondary school only	52.3	23.5	83.0	11.32 (.50,.67)	0.001
Unemployed	39.6	60.1	19.4	− 7.84 (− .4,9,− .31)	0.001
Job in informal sector	60.6	52.4	81.4	5.58 (.19, .38)	0.001
Receives money from family/friends	7.7	1.1	15.0	4.92 (.08, .19)	0.001
Medical Insurance	26.4	38.4	15.2	− 4.86 (− .31,− .14)	0.001

**Table 2:** HIV risk behaviors

	Total *N* = 363	El Paso *N* = 187	Cd. Juárez *N* = 176	Z (95% CI of the difference)	*P*-value
*Percent unless indicated*					
Condomless sex with more than 1 partner	55.3	54.5	56.3	.32 (− .08, .11)	0.744
More than 1 STI	35.8	34.2	37.5	.65 (− .06, .13)	0.515

**Table 3:** Micro-level factors (injecting environment at drug use site most frequented to use drugs and using drugs with others)

Injecting environment	Total *N* = 363	El Paso *N* = 187	Cd. Juárez *N* = 176	Z (95% CI of the difference)	*P*-value
*Percent unless indicated*					
Injects in public spaces	29.4	41.6	17.1	− 5.79 (− .33, − .15)	0.001
Injects in shooting galleries	26.9	8.0	46.9	8.14 (.29, .46)	0.001
Drugs available for purchase	29.7	40.1	27.7	− 2.30 (− .22,− .01)	0.02
Polysubstance use	77.1	77.9	86.3	2.01 (.002,.16)	0.04
Syringe sharing	34.9	36.7	48.2	2.0 (.002, .22)	0.04
Sex for drug exchanges	29.2	31.1	39.1	1.45 (− .02, .18)	0.145
Injected with other people	80.7	83.2	80.3	− 0.71 (− .10, .05)	0.477

**Table 4:** Macro-level factors (manifestations of sociocultural and economic context, harm reduction policies, drug market economy, criminal justice system, and stigma)

	Total *N* = 363	El Paso *N* = 187	Cd. Juárez *N* = 176	Z (95% CI of the difference)	*P*-value
*Percent unless indicated*					
Ever been in prison	76.3	82.3	71.7	− 2.38 (− .19, − .01)	0.017
Ever been subjected to policing	40.7	17.6	65.3	9.24 (.38, .56)	0.001
Involved in drug selling	12.1	19.8	8.2	− 2.65 (− .19, − .03)	0.004
Lived in another country	28.9	21.4	36.9	3.26 (.06,.24)	0.001
Lived in another state	52.3	64.0	43.0	− 3.93 (− .30,− .10)	0.001
Drug use stigma	77.6	80.4	79.8	− .158 (− .09, .07)	0.874
Unhoused	30	34.6	26.4	− 1.65 (− .17, .01)	0.097

**Table 5:** Generalized estimating equations for syringe sharing in El Paso (*N* = 187)

Parameter	*β*	*SE*	*t*	*OR*	95% CI	*p*
Intercept	.49	1.8	.64	.61	.01, 21.68	.78
Gender^a^	− .59	.38	1.52	1.80	.84, 3.88	.12
Education^b^	.35	.36	.97	1.42	.69, 2.93	.33
Marital status^c^	− .16	.34	− .46	.85	.43, 1.68	.64
History of U.S. State-Immigration^d^	.03	.42	.09	1.03	.45, 2.39	.92
Injecting in a Public Space^d^	− .28	.35	− .80	.75	.37, 1.50	.42
Drugs sold at drug use site^d^	1.05	.35	2.95	2.87	1.41, 5.81	.004
History of incarceration^d^	.19	.47	.40	1.21	.47, 3.10	.40
Involved in drug selling^d^	− .14	.44	− .32	.86	.36, 2.06	.74

All variables are dichotomous

^a^0 = male versus 1 = female; ^b^0 = other category versus 1 = high school only; ^c^0 = other category versus 1 = single; ^d^0 = absence of the category

As shown in Table 1, approximately 24.6% of the EP study sample were women, while in CJ only 15% of the sample were women, *p* < .05. In CJ, 39.2% of the participants reported being in a stable relationship compared with less than one quarter (22.6%) in EP, *p* < .01. Other significant differences emerged such as a greater proportion of individuals in CJ (83%) having elementary school only compared to EP (23.5%), and a greater proportion of individuals in EP having completed high school, *p* < .01. In addition, 19.4% of CJ participants reported to be unemployed compared to EP (60.1%), *p* < .01. However, a higher proportion of individuals residing in CJ (81.4%) reported being employed in the informal sector compared to individuals residing in EP (52.4%), *p* < .01. Moreover, in EP only one percent of the participants reported to have received financial support from a friend or family member, while in CJ 15.2% have, *p* < .01. Lastly, in CJ only 15% of the participants reported to have medical insurance ,while in EP nearly 40% of the participants have some form of medical insurance, *p* < .01.

Table 2 presents differences across sites in condomless sex and history of STIs. As Table 2 indicates, similar proportions across the cities reported engaging in sexual risk behaviors including engagement in condomless sex with multiple sex partners and ever been diagnosed with more than 1 STI.

Table 3 presents differences and similarities in micro-level factors. As Table 3 indicates, EP participants were significantly more likely to report injecting in public spaces (41.6%) compared to only 17.1% in CJ, *p* < .01. In CJ, almost half (46.9%) of the participants report injecting in a shooting gallery compared to 8% of EP participants, *p* < .01. Moreover, differences emerged in the dynamics of risk present at the drug use site most frequented. Specifically, a greater proportion of individuals in EP (40%) reported the availability of drugs for purchase at the drug use site most frequented than participants in CJ (28%), *p* < .05. In addition, a larger proportion of participants in CJ (48.2%) compared with EP (36.7%) reported using drugs in a site where syringes are shared, *p* < .05. Moreover, participants in CJ (86%) were more likely to report polysubstance use at the drug use site most frequented than their counterparts in EP (78%), *p* < .05. Lastly, no significant differences emerged in frequency of injecting with other people and exchanging sex for drug exchanges at the drug use site most frequented.

Table 4 presents differences and similarities in macro-level factors. As Table 4 indicates, large proportions of participants in both cities reported ever being incarcerated; however, the proportion was significantly larger in EP (82.3%) compared to CJ (71.7%), *p* < .05. A greater proportion of participants in CJ (65.3%) reported experiencing harsh policing compared to 17.6% in EP, *p* < .01, while a greater proportion of participants in EP (19.8%) reported being involved in drug selling compared to 8.2% of CJ participants, *p* < .01. In terms of migration history, participants in both sites were highly mobile, but a greater proportion of participants from CJ (37%) reported to have lived outside the country compared with 21% of EP participants, *p* < .01. Additionally, a greater proportion of EP participants (64%) reported movement between states within the country of residence compared with 43% of CJ participants, *p* < .01. Lastly, no significant differences were observed in substance use stigma and housing as similar proportions of participants perceived a high degree of substance use stigma and were unhoused across the two cities.

Results of the GLMM equation for EP, presented in Table 5, indicate using drugs in a drug use site where drugs are sold is associated with increased syringe sharing (OR = 2.62, *p* = .007). Results for CJ, presented in Table 6, indicate injecting drugs in a shooting gallery is associated with increased syringe sharing (OR = 2.87, *p* = .004).

**Table 6:** Generalized estimating equations for syringe sharing in Ciudad Juarez (*N* = 176)

Parameter	*β*	*SE*	*t*	*OR*	95% CI	*p*
Intercept	− .27	.49	− .54	.76	.28, 2.04	.58
Education^a^	.34	.35	.97	1.41	.70, 2.83	.33
Marital status^b^	.53	.36	1.46	1.70	.83, 3.48	.14
History of immigration^c^	− .51	.37	− 1.36	.60	.28, 1.25	.17
Policing^c^	.71	.38	1.85	2.04	.95, 4.39	.06
Injecting in a shooting gallery^c^	.96	.35	2.73	2.62	1.30, 5.28	.007
Polydrug use at drug use site^c^	− .20	.46	− .44	.84	.32, 2.03	.65
Receive support from family^c^	− .18	.34	− .53	.83	.41, 1.65	.59

All variables are dichotomous

^a^0 = other category versus 1 = partnered; ^b^0 = other category versus 1 = elementary school only; ^c^0 = absence of the category

## Discussion

The purpose of the study was to understand the factors that influence HIV risk among PWID residing in an under resourced binational setting characterized by high cross-country population mobility. Our aim was to understand similarities and differences between PWID residing in either city to inform potential policy and behavior change interventions to ameliorate HIV risk. Results of comparisons indicated that similar proportions of participants in both cities reported engaging in HIV risk behaviors such as condomless sex with multiple partners and reported having a history of STIs. In addition, similarities emerged in other characteristics of the drug use setting (micro-level factors) such as injecting with other people and engaging in sex for drug exchanges at the drug use site most frequented. Moreover, similar proportions were affected by substance use stigma and housing instability. These similarities underscore the need to implement multilevel interventions binationally to promote engagement in harm reduction and the dismantling of structural level factors such as stigma. In spite of the two countries having diverse policy environments that distinctly shape acceptance of harm reduction, stigma toward substance use is a powerful deterrent in both countries that limits the allocation of resources to combat the negative public health consequences of injection drug use including access to harm reduction services and housing. Differences emerged in terms of socioeconomic factors with a greater proportion of participants in CJ having completed elementary school only while a lower proportion was more likely to be unemployed compared to EP. This counterintuitive finding may be explained by socio-contextual differences across the cities that may afford engagement in different income generating activities. CJ has large public markets where second-hand goods are sold, for example. As a result, PWID may have increased opportunities of being informally employed in CJ compared to EP. However, similar proportions reported housing instability, a structural level factor that consistently emerges as one of the most important factors to target in structural-level interventions. A concerted binational multilevel effort is needed that can target factors operating at multiple levels. For example, a binational multilevel intervention consisting of a social marketing campaign to reduce stigma combined with a microenterprise intervention to reduce economic vulnerability and peer-led strategies to increase access to harm reduction resources may be impactful.

Regarding differences in macro-level factors such as involvement with the criminal justice system, findings indicated that a larger proportion of individuals in CJ report experiencing harsh policing while a slightly larger proportion of individuals residing in EP has been incarcerated. The risk environment framework conceives of incarceration and imprisonment as a structural factor and risk environment on its own as incarceration predicts HIV acquisition [40]. This finding also underscores the need to develop and test interventions for populations that have a history of incarceration and to build capacity in law enforcement in CJ through local binational law enforcement trainings. Past efforts done to train law enforcement in Mexican cities along the US–Mexico border such as Tijuana appear to be a promising strategy to reduce human right violations of PWID [41]. Binational agreements that promote collaboration between law enforcement agencies in US–Mexico border sister cities may prove fruitful to build capacity to reduce the harm of substance use in binational communities. Moreover, a greater proportion of individuals in EP indicated consuming drugs at sites where drugs are sold and in results of GLMM equations this factor alone emerged as significantly related to increased syringe sharing. This finding points to the need to implement harm reduction strategies in sites where drugs are sold. In our experience, delivering harm reduction at places where drugs are sold is challenging, as sellers are powerful gatekeepers. Conversely, in CJ, a greater proportion of individuals indicated using drugs at sites where multiple substances are consumed. Future research is warranted to understand how harm reduction interventions could be delivered at drug use sites characterized by such dynamics of drug use risk.

Results indicated that a greater proportion of individuals in EP report injecting in public spaces compared to individuals in CJ where a greater proportion indicates injecting in shooting galleries, and again, this factor alone was associated with syringe sharing in CJ. Research informed by the risk environment framework indicates that the features of the injecting environment are a micro-level factor that may inhibit or promote health harms [16]. Injecting in a shooting gallery may promote syringe sharing as other PWID are likely to be around compared to injecting in a public space such as public restroom. Although the reasons why a greater proportion of individuals in CJ report injecting in a shooting gallery compared to EP participants remains elusive, this finding may be explained by the greater availability of shooting galleries observed in CJ compared to EP. This finding may suggest that potential differences in the socio-context of injection drug use remain misunderstood as there is a dearth of research exploring how socio-contextual-level factors influence the availability of places to use drugs. This finding has implications for the manner in which street outreach and delivery of harm reduction intervention are planned across the two cities.

In terms of demographic characteristics, PWID recruited in CJ are more likely to be partnered which has implications for the types of interventions that could be more effective to reduce harm. Couples-based interventions may be more likely to promote sustainable behavior change.

Other findings indicated that the majority of participants living in EP were more likely to have lived or worked in another state (within the USA). Mobility has been identified as an important driver of Mexico’s HIV epidemic, especially among migrant men who are more likely to have sex with other men, and to pay for sex with men and women, compared to non-migrants [3, 12, 42–44]. There is limited research regarding how mobility across US states may influence HIV risk. Regarding criminal justice involvement, similar high proportions of PWID had a history of incarceration, with a significant difference in proportions across the cities where individuals residing in EP were more likely to have history of incarceration. Similarly, a larger proportion of PWUD residing in EP report being involved in the drug market economy.

The lower proportion of women IDUs recruited in this study was similar to a more recent study conducted with PWID in CJ as well as Tijuana [3, 45]; however, the small number of women may have limited our ability to capture important patterns in behaviors that have been associated with HIV infection. There is growing evidence that drug use is now on the rise among women [46]; however, the extent to which drug use has become entrenched in drug use culture on the US–Mexico border among both men and women, may be a harbinger of rising rates of HIV and STIs.

Our study has limitations. One important limitation of our study is the use of a non-random sample, which limits generalizability. However, our findings can pave the way for future research among binational contexts to inform interventions that are likely to have a sustained effect in highly mobile populations. Another limitation is that variables that emerged as significantly different in city pairwise comparisons were entered into the multivariate analysis. Although this approach may also limit generalizability of findings, the postulation of hypothesis regarding differences between the two cities was not possible due to the dearth of prior research comparing the cities.

## Conclusions

This preliminary CBPR study was conducted as the first phase of Project Encuentro, a binational collaboration between community-based organizations, researchers, and peers to adapt and implement a harm reduction intervention with the ultimate goal of assessing and intervening on the HIV risk of persons who use drugs in the twin cities of CJ/EP [34]. Shared micro- and macro-level factors among the two sister cities including *injecting with other people*, *engaging in syringe sharing at drug use sites frequented*, *felt substance use stigma*, and *housing instability* highlight the need for binational, multilevel harm reduction strategies. Findings from this comparative cross-sectional study informed phase II of Project Encuentro which consisted in intervention adaptation implementation, and testing to increase access to rapid HIV testing, to reduce HIV risk behaviors through a peer network behavioral intervention, and community-wide events targeting structural factors affecting HIV risk among CJ/EP. Findings from this cross-sectional study and subsequent CBPR intervention can serve as a foundation to advance regional harm reduction policies and practices.

## Data Availability

Data will be shared upon request.

## Abbreviations

PWID: People who inject drugs
HIV: Human immunodeficiency virus
STIs: Sexually transmitted infections
MSM: Men who have sex with men
PWUD: People who use drugs
CJ/EP: Ciudad Juárez, Chihuahua-Mexico and El Paso, Texas-U.S.,
Cd.: Ciudad
STROBE: Strengthening the Reporting of Observational Studies in Epidemiology

## References

[1] Bourgois P, Holmes SM, Sue K, Quesada J (2017). Structural vulnerability: operationalizing the concept to address health disparities in clinical care. Acad Med J Assoc Am Med Coll.

[2] Chakrapani V, Newman PA, Shunmugam M, Dubrow R (2011). Social-structural contexts of needle and syringe sharing behaviours of HIV-positive injecting drug users in Manipur, India: a mixed methods investigation. Harm Reduct J.

[3] Ramos R, Ferreira-Pinto JB, Brouwer KC, Ramos ME, Lozada RM, Firestone-Cruz M (2009). A tale of two cities: social and environmental influences shaping risk factors and protective behaviors in two Mexico–US border cities. Health Place.

[4] Rhodes T (2009). Risk environments and drug harms: a social science for harm reduction approach. Int J Drug Policy.

[5] Rhodes T, Wagner K, Strathdee SA, Shannon K, Davidson P, Bourgois P, O’Campo P, Dunn JR (2012). Structural violence and structural vulnerability within the risk environment: theoretical and methodological perspectives for a social epidemiology of HIV risk among injection drug users and sex workers. Rethinking social epidemiology: towards a science of change [Internet].

[6] Riley ED, Gandhi M, Hare C, Cohen J, Hwang S (2007). Poverty, unstable housing, and HIV infection among women living in the United States. Curr HIV/AIDS Rep.

[7] Auerbach JD, Parkhurst JO, Cáceres CF (2011). Addressing social drivers of HIV/AIDS for the long-term response: conceptual and methodological considerations. Glob Public Health.

[8] Becker MH (1974). The health belief model and sick role behavior. Health Educ Monogr.

[9] Janz NK, Becker MH (1984). The health belief model: a decade later. Health Educ Q.

[10] Ajzen I, Fishbein M (1980). Understanding attitudes and predicting social behavior.

[11] Rhodes T (2002). The ‘risk environment’: a framework for understanding and reducing drug-related harm. Int J Drug Policy.

[12] Rhodes T, Quirk A (1998). Drug users’ sexual relationships and the social organisation of risk: the sexual relationship as a site of risk management. Soc Sci Med.

[13] Degenhardt L, Mathers B, Vickerman P (2010). Prevention of HIV infection for people who inject drugs: why individual, structural, and combination approaches are needed. Lancet.

[14] Rhodes T, Singer M, Bourgois P, Friedman SR, Strathdee SA (2005). The social structural production of HIV risk among injecting drug users. Soc Sci Med.

[15] Strathdee SA, Hallet TB, Bobrova N (2010). HIV and risk environment for injecting drug users: the past, present, and future. Lancet.

[16] Rhodes T, Kimber J, Small W (2006). Public injecting and the need for ‘safer environment interventions’ in the reduction of drug-related harm. Addiction.

[17] Miller CL, Firestone M, Ramos R (2008). Injecting drug users’ experiences of policing practices in two Mexican-US border cities: public health perspectives. Int J Drug Policy.

[18] Strathdee SA, Fraga WD, Case P (2005). Vivo para consumirla y la consumo para vivir [I live to inject and inject to live]: high-risk injection behaviors in Tijuana Mexico. J Urban Health.

[19] Strathdee SA, Lozada R, Pollini RA (2008). Individual, social, and environmental influences associated with HIV infection among injection drug users in Tijuana Mexico. J Acquir Immune Defic Syndr.

[20] Brouwer KC, Lozada R, Cornelius WA, et al. Deportation along the U.S.-Mexico border: its relation to drug use patterns and accessing care. J Immigr Minor Health. 2008. 10.1007/s10903-008-9119-5 PMC3154615 18247117

[21] INEGI. México en cifras: Juárez, Chihuahua (08037) [Internet]. INEGI; Mexican National Institute of Geography and Statistics. Instituto Nacional de Estadística y Geografía. INEGI; 2020 [cited 2021 Oct 26]. Available from: https://www.inegi.org.mx/app/areasgeograficas/.

[22] U.S. Census Bureau. 2020 Census - Census Block Maps [Internet]. 2020. Available from: https://www.census.gov/geographies/reference-maps/2020/geo/2020-census-block-maps.html.

[23] Office of Global Affairs. The U.S.-Mexico Border Region [Internet]. HHS.gov. 2017. Available from: https://www.hhs.gov/about/agencies/oga/about-oga/what-we-do/international-relations-division/americas/border-health-commission/us-mexico-border-region/index.html.

[24] Paso del Norte Health Foundation. Healthy Paso del Norte: Community Dashboard [Internet]. Healthy Paso del Norte Community Dashboard: Far West Texas, Southern New Mexico, Ciudad Juarez. 2021. Available from: http://www.healthypasodelnorte.org/.

[25] U.S. Census Bureau. U.S. Census Bureau QuickFacts: El Paso County, Texas; Texas; United States [Internet]. 2020. Available from: https://www.census.gov/quickfacts/fact/table/elpasocountytexas,tx,US/RHI725217#viewtop.

[26] U.S. Department of Transportation. Border Crossing Entry Data: Research and Statistics [Internet]. The Bureau of Transportation Statistics (BTS) Border Crossing Data. 2022. Available from: https://data.bts.gov/Research-and-Statistics/Border-Crossing-Entry-Data/keg4-3bc2.

[27] U.S. Department of Health and Human Services. Health Insurance Coverage and Access to Care Among Latinos: Recent Trends and Key Challenges [Internet]. Washington, DC: Office of the Assistant Secretary for Planning and Evaluation; 2021 Oct. Report No.: HP-2021-2. Available from: https://aspe.hhs.gov/reports/health-insurance-coverage-access-care-among-latinos.

[28] NIDA. Trends and Statistics: Overdose Death Rates [Internet]. National Institute on Drug Abuse. 2022. Available from: https://nida.nih.gov/research-topics/trends-statistics/overdose-death-rates.

[29] Cravioto P. La magnitud y naturaleza del problema de la heroina en Ciudad Juarez, Chihuahua [Internet]. [Mexico, D.F.]: Universidad Nacional Autonoma de Mexico; 2003. Available from: http://132.248.9.195/ppt2002/0316322/Index.html.

[30] INEGI. Poblacion, Hogares y Vivienda [Internet]. INEGI; Mexican National Institute of Geography and Statistics. Instituto Nacional de Geografia y Estadística. INEGI; 2013 [cited 2022 May 14].Available from http://www3.inegi.org.mx/sistemas/temas/default.aspx?s=est&c=17484.

[31] Lechuga J, Ramos R, Dickson-Gomez J, et al. Institutional violence from pólice militarization and drug cartel wars as a ‘big event’ and its influence on drug use harms and HIV risk in people who use drugs on the US-Mexico border. Manuscript submitted for publication.10.1016/j.drugpo.2023.10412537499305

[32] Resendiz, J. Juarez Murders up 42% Despite Quarantine. Border Crime. 2020, June 13 [cited 2023 May 14]. Available at: https://www.borderreport.com/hot-topics/border-crime/juarez-murders-up-42-despite-quarantine-police-blame-violence-on-gangs/.

[33] Lechuga J, Esparza O, Aleman J, et al. The perpetuation of HIV risk among Central American and Cuban immigrants at the US-Mexico border: a transnational perspective. Manuscript submitted for publication.

[34] Lechuga J. Adaptation and Implementation of Project Encuentro in the U.S.-Mexico Border [Internet]. clinicaltrials.gov; 2017 Sep [cited 2021 Oct 24]. Report No.: NCT03293875. Available from: https://clinicaltrials.gov/ct2/show/NCT03293875.

[35] von Elm E, Altman DG, Egger M, Pocock SJ, Gøtzsche PC, Vandenbroucke JP (2008). The strengthening the reporting of observational studies in epidemiology (STROBE) statement: guidelines for reporting observational studies. J Clin Epidemiol.

[36] Heckathorn DD (1997). Respondent-driven sampling: a new approach to the study of hidden populations studying hidden populations. Soc Probl [Internet].

[37] Ramos RL, Ferreira-Pinto JB, Rusch MLA, Ramos ME (2010). Pasa la Voz (Spread the Word): using women’s social networks for HIV education and testing. Public Health Rep.

[38] Bolker BM. Linear and generalized linear mixed models. In: Ecological Statistics [Internet]. Oxford: Oxford University Press; 2015 [cited 2022 Apr 9]. Available from: https://www.universitypressscholarship.com/.

[39] Bono R, Alarcón R, Blanca MJ (2021). Report quality of generalized linear mixed models in psychology: a systematic review. Front Psychol [Internet].

[40] Choopanya K, Des Jarlais DC, Vanichseni S (2002). Incarceration and risk for HIV infection among injection drug users in Bangkok. J AIDS.

[41] Arredondo J, Strathdee SA, Cepeda J, Abramovitz D, Artamonova I, Clairgue E (2017). Measuring improvement in knowledge of drug policy reforms following a police education program in Tijuana, Mexico. Harm Reduct J.

[42] Rachlis B, Brouwer KC, Mills EJ, Hayes M, Kerr T, Hogg RS (2007). Migration and transmission of blood-borne infections among injection drug users: understanding the epidemiologic bridge. Drug Alcohol Depend.

[43] Ramos R (1990). Migratory patterns and the spread of HIV across the Mexico-United States Border. Salud Front.

[44] Strathdee SA, Philbin MM, Semple SJ, Pu M, Orozovich P, Martinez G (2008). Correlates of injection drug use among female sex workers in two Mexico-U.S. border cities. Drug Alcohol Depend.

[45] Strathdee SA, Lozada R, Ojeda VD, Pollini RA, Brouwer KC, Vera A (2008). Differential effects of migration and deportation on HIV infection among male and female injection drug users in Tijuana, Mexico. PLoS ONE.

[46] Shedlin M, Ramos R. Methamphetamine use among male and female populations. In: XVIII International AIDS Conference; Mexico City, Mexico 2008.

